# Nanomaterials-Enhanced Electrochemical Biosensors for Epithelial Cancer Diagnosis: Recent Advances

**DOI:** 10.3390/bios15120766

**Published:** 2025-11-22

**Authors:** Matías Regiart, Alba M. Gimenez, Francisco G. Ortega, Germán E. Gómez, Juan Sainz, Gonzalo R. Tortella, Martín A. Fernández-Baldo

**Affiliations:** 1 Instituto de Química de San Luis, Facultad de Química, Universidad Nacional de San Luis, INQUISAL (UNSL—CONICET), Ejército de los Andes 950, San Luis D5700BWS, Argentina; regiart@unsl.edu.ar; 2 Department of Immunology, Institute of Biomedical Sciences, Universidade de São Paulo (USP), São Paulo 05508-220, Brazil; albamarinagimenez@gmail.com; 3 GENYO, Centre for Genomics and Oncological Research, Pfizer/University of Granada/Andalusian Regional Government PTS, Granada, Avenida de la Ilustración, 114, 18016 Granada, Spain; gabriel.ortega@genyo.es; 4 IBS Granada, Institute of Biomedical Research, Avenida de Madrid 15, 18012 Granada, Spain; 5 Instituto de Investigaciones en Tecnología Química (INTEQUI), Departamento de Química, Universidad Nacional de San Luis (UNSL), CONICET, Ejército de los Andes 950, San Luis D5700BWS, Argentina; germangomez1986@gmail.com; 6 Genomic Oncology Area, GENYO, Centre for Genomics and Oncological Research: Pfizer/University of Granada/Andalusian Regional Government, PTS, 18016 Granada, Spain; jsainz@ugr.es; 7 Instituto de Investigación Biosanataria IBs, Granada, 18012 Granada, Spain; 8 Consortium for Biomedical Research in Epidemiology and Public Health (CIBERESP), University of Barcelona, 08908 Barcelona, Spain; 9 Department of Biochemistry and Molecular Biology I, University of Granada, 18012 Granada, Spain; 10 Centro de Excelencia en Investigación Biotecnológica Aplicada al Medio Ambiente (CIBAMA), Facultad de Ingeniería y Ciencias, Universidad de La Frontera, Av. Francisco Salazar 01145, Temuco 4811230, Chile; 11 Departamento de Ingeniería Química, Facultad de Ingeniería y Ciencias, Universidad de La Frontera, Av. Francisco Salazar 01145, Temuco 4811230, Chile

**Keywords:** biosensor, electrochemical, nanomaterial, tumor biomarker, cancer diagnosis, real samples

## Abstract

In recent years, the interest in electrochemical biosensors has been constantly growing for epithelial cancer diagnosis and prognosis. The incorporation of the different nanomaterials as metal nanoparticles, magnetic nanoparticles, carbon nanomaterials, Metal–Organic Frameworks (MOFs), and nanocomposites, along with specific monoclonal antibodies, and nucleic acids (aptamers) has improved both sensitivity and specificity in these methodologies. In this review, we have presented examples of electrochemical biosensors for the determination of different epithelial cancer biomarkers. Based on numerous reports in the recent literature, we highlight the use of single and multiplexed analytical platforms for the quantification of epithelial cancer biomarkers. In addition, we outline potential development pathways, current challenges, and future prospects in the field of electrochemical immuno-, apta-, and genosensors.

## 1. Introduction

Cancer is one of the most significant global health challenges today, recognized as a leading cause of morbidity and mortality worldwide. Epithelial cancer is particularly relevant as it encompasses a wide range of common cancers, including breast, prostate, colon, colorectal, lung, ovarian, stomach, pancreatic, and thyroid cancers [1,2]. With the highest mortality rate among all cancer types, epithelial cancer has become a major global threat. This high mortality is primarily due to the lack of specific early-stage symptoms, which often leads to a late diagnosis, typically after the cancer has already metastasized [3,4].

The traditional diagnosis and prognosis of cancer rely on identifying specific tumor markers for each cancer type, which must be correlated with the patient’s clinical data [5,6]. Consequently, early diagnosis and treatment are crucial for improving patient mortality rates and quality of life [7,8]. This has created an urgent clinical need for economical and effective methods for the early diagnosis of these cancers [9]. The rapid analysis and determination of specific epithelial cancer biomarkers in biological samples such as blood, urine, serum, saliva, or extracellular vesicles (EVs) are essential for early screening, understanding disease progression, and guiding treatment prognosis [8,9].

However, classic diagnostic methods, including immunohistochemistry, histopathology, enzyme-linked immunosorbent assay (ELISA), and radioimmunoassay, are often slow, expensive, and require highly trained personnel [10]. For these reasons, developing sensitive, practical, and affordable methods for detecting specific epithelial biomarkers is critical for advancing early cancer diagnosis [11]. Electrochemical techniques offer a relevant alternative due to their inherent sensitivity, selectivity, rapid response times, and potential for miniaturization [12,13].

In recent years, a wide range of sophisticated electrochemical biosensors has been developed for application in oncology. These integrated analytical devices convert the information from the molecular recognition process between a bioreceptor and the target analyte (the cancer biomarker) into a quantifiable signal [14,15]. The type of bioreceptor used, such as antibodies, antigens, oligonucleotides, or aptamers, also defines the biosensors category, leading to different types like immunosensors, genosensors, and aptasensors [16].

These devices rely on several electrochemical detection techniques, among which cyclic voltammetry (CV), differential pulse voltammetry (DPV), square-wave voltammetry (SWV), and electrochemical impedance spectroscopy (EIS) are the most commonly used. The resulting electronic signals are then correlated with the concentration of a specific biomarker, providing a diagnostic result [17].

These sensors are characterized by their multiplexing capabilities, high sensitivity, excellent selectivity, fast detection, portability, and the potential for integration with advanced electronics [18,19]. To achieve low limits of detection, researchers often utilize various strategies, such as incorporating nanomaterials as immobilization platforms for biomolecules (DNA, RNA, antigens, antibodies, or aptamers) [19]. Another effective strategy is to combine polymers and nanoparticles with conjugated structures, which synergistically enhances biosensor performance [20].

This review aims to summarize the latest electrochemical biosensors developed for specific epithelial cancer biomarkers determination, and provides valuable information for scientists working on developing new analytical devices with enhanced selectivity and specificity for cancer diagnosis and prognosis. We present examples that illustrate the use of diverse nanomaterials and bioreceptors, including the detection of newly identified biomarkers, together with detailed insights into design strategies, key characteristics, and the types of samples evaluated for both single and multiplex biomarker detection. The following sections include a brief introduction to electrochemical biosensors, followed by a discussion of recent publications, including cancer antigens, carcinoembryonic antigens, prostate-specific antigens, RNA, DNA, EVs and cells, and other relevant epithelial biomarkers. Finally, we discuss the challenges and prospects in this important field.

## 2. Electrochemical Biosensors

**Figure 1:**
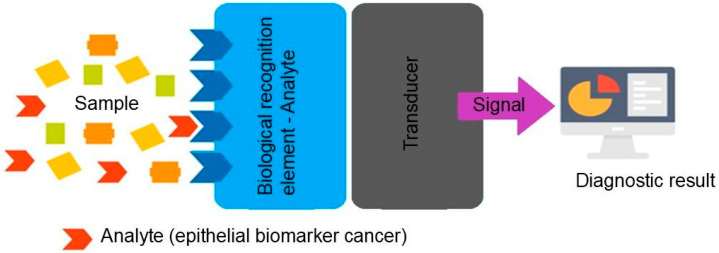
Schematic representation of a biosensor. Figure made by the authors.

A biosensor is a compact analytical device that integrates a biological recognition element (such as an antigen, antibody, DNA, RNA, enzyme, or aptamer) with a signal transducer, typically an electrochemical or optical detector. This design allows the biosensor to recognize specific target molecules and translate this interaction into a measurable signal (Figure 1) [1,2,3].

These sensors are typically constructed with three main components: a working electrode, a reference electrode, and an auxiliary electrode. The working electrode interacts directly with the analyte during detection, while the other two electrodes maintain a stable potential and complete the electrical circuit, respectively [4]. These features make electrochemical biosensors highly suitable for addressing diagnostic needs, particularly for specific epithelial cancers. In this field, biomarkers are crucial for early detection, guiding treatment selection, and assessing prognosis. Electrochemical biosensors offer significant advantages over traditional methods for detecting these biomarkers, including high sensitivity, selectivity, stability, and low cost [2]. By utilizing diverse recognition elements and advanced materials like nanomaterials, electrochemical biosensors hold immense promise for increasing detection sensitivity, paving the way for groundbreaking diagnostic and therapeutic strategies in epithelial cancer [2,3,4]. Nanomaterials have been used for electrode modification or for the immobilization of biomolecules, leading to the development of biosensors with low detection limits [3,4].

## 3. Epithelial Cancer Biomarkers

### 3.1. Cancer Antigen (CA)

The epithelial cancer biomarkers are biological molecules that enable early detection, diagnosis, prognosis, and monitoring of treatment in cancer patients. They are clinically significant because they help identify tumor type and status, predict response to specific therapies, assess disease progression, and detect recurrence. Their use enhances the precision of personalized therapeutic decisions and can optimize treatment efficacy and patient follow-up during and after medical intervention [21,22].

Cancer antigen 125 (CA125) is a crucial biomarker in ovarian cancer diagnosis, where elevated serum levels (>35 U mL^−1^) are a key risk indicator. The need for early, sensitive, and reliable detection of CA125 and other biomarkers has driven the development of advanced biosensors [22].

In this context, AuNPs and carbon-based materials, such as reduced graphene oxide (rGO) and carbon nanotubes, are the most frequently used nanomaterials. These are chosen for their synergistic properties, which boost conductivity, increase the active surface area, and optimize the immobilization of recognition elements. Additionally, innovative platforms like metal–organic frameworks (MOFs) and natural product hybrids are emerging to create 3D architectures that amplify the signal. Maximum sensitivity is achieved using advanced voltammetric techniques such as DPV or SWV, which reduce background noise to reach LODs significantly below clinical thresholds. The reliability of these biosensors is confirmed through their validation in real serum samples, establishing them as a promising technology for point-of-care (POC) clinical cancer diagnosis. In this section, we highlight selected examples of sensing devices (see Table 1) that demonstrate how the incorporation of nanomaterials not only increases versatility but also addresses common limitations of conventional approaches, such as stability and reproducibility.

For instance, Yilmaz et al. [21] reported the first fabrication of a rapid, low-cost, disposable, and label-free CA125 immunosensor achieved by modifying poly toluidine blue (PTB) synthesized in a green solvent with AuNPs. The synergistic effect of PTBDES and AuNPs enhanced both the electrode’s electrochemical properties and the overall sensing performance. The immunosensor exhibited excellent stability during storage and regeneration, as well as high selectivity in the presence of other tumor markers. CA125 detection in blood serum yielded recoveries with < 5% error, confirming its reliability. Owing to its simplicity, reproducibility, and strong analytical performance, this disposable CA125 immunosensor represents a promising candidate for point-of-care clinical testing of ovarian cancer biomarkers. Similarly, another CA125 immunosensor, based on amino-functionalized carbon nanotube on screen-printed carbon electrode Ti_3_C_2_Tx/NH_2_-CNT-SPCE, exhibited rapid electron transfer, signal amplification, and enhanced stability via chitosan [22]. Compared with the AuNPs/PTB system, this approach offered a wider detection range (1 –500 U mL^−1^) with low LOD (1 mU mL^−1^), good selectivity, and reliable performance in clinical serum, demonstrating strong potential for ovarian cancer diagnostics. Notably, the clinical cut-off value for CA125 is ~35 U mL^−1^ in serum, which means that the reported LOD is well below the threshold required for clinical relevance. Guo et al. [23] developed Mn-doped ZnO nanorods functionalized with Au-NPs (ITO-Mn/ZnO-Au). The composite exhibited enhanced conductivity, charge transfer, and electrocatalytic activity, enabling efficient aptamer immobilization. The biosensor achieved a wide linear range (0.002–3 ng mL^−1^), ultralow LOD (0.5 pg mL^−1^), good reproducibility (RSD = 3.5%), and high recovery (97.2–105%) in patient serum, highlighting its promise for clinical applications. In addition, traditional materials such as spinel NiFe_2_O_4_ magnetic nanoparticles, synthesized via the hydrothermal method, were employed as electrode material for CA125 detection [24]. The resulting immunosensor exhibited a low LOD (<8.5 U mL^−1^), good reproducibility, and strong selectivity against serum proteins and other biomarkers.

Recent advances have explored the use of natural product-derived materials as functional sensing platforms for tumor biomarkers. A notable example is the development of onion oil-based organic–inorganic hybrids (ONOHs) for CA-125 detection in serum [25]. These hybrids gels, formed by physical or chemical cross-links of synthetic or naturally derived molecules, integrate onion oil within a porous 3D network by using agar, glycerol, and glutaraldehyde crosslinker through a free radical polymerization process, which significantly enhances electrocatalytic activity and reduces charge transfer resistance. Among the reported systems, ONOH-3 exhibited the best electrochemical performance, achieving a low detection limit (0.805 μU mL^−1^), broad linear ranges (0.5–10 and 10–300 ng mL^−1^), and high selectivity against potential interferents in serum. The improved sensitivity and stability of ONOH-3 highlight the promise of onion oil-based hybrids as cost-effective, biocompatible sensing materials with strong potential for clinical applications in ovarian cancer diagnostics.

A recent strategy for tumor marker detection combined rGO-TEPA/ZIF-67@ZIF-8/Au substrates (ZIF = Zeolitic Imidazol Frameworks; rGO-TEPA = reduced graphene oxide-tetraethylenepentamine) with AuPdRu trimetallic nanoparticles to construct a sandwich-type electrochemical immunosensor for CA72-4 [26]. The system leverages core–shell ZIF nanoparticles, Au-NPs, and rGO-TEPA to achieve high catalytic activity, a wide linear range (0.0001–1000 U mL^−1^), and low detection limits. Another example of an MOF platform employed for CA125 detection is the ratiometric electrochemical immunosensor based on 3DrGO/MWCNTs-Thi and UiO-66-Fc [27]. The 3D rGO/MWCNTs framework enhanced conductivity and Ab1 loading, while UiO-66-NH_2_ offered high surface area for Fc-COOH/Ab2 immobilization, boosting signal amplification.

Another example of graphene-derived nanomaterial proposed as an immunosensor is the nitrogen-doped graphene oxide decorated platinum cobalt (NGO-PtCo) -modified carbon fiber aptasensor for CA15-3, which showed a low LOD (4.1 × 10^−2^ U mL^−1^), high sensitivity, and excellent specificity in human serum, with 92–100% recovery. Despite requiring further clinical validation and cost-optimization, it represents a promising platform for breast cancer diagnostics [28]. Regarding the detection of CA15-3, Oliveira and colleagues reported a novel MIP (molecularly imprinted polymers) prepared via additional activators and reducing agents (SARA) through atom transfer radical polymerization (ATRP), enabling highly reproducible and sensitive detection, outperforming conventional radical polymerization. The biosensor achieved a wide linear range down to 0.001 U mL^−1^ (10× better than conventional methods). It showed excellent selectivity and reproducibility, attributed to the controlled synthesis of PAAm-co-PMBAm (copolymer of acrylamide and N,N′-methylenebisacrylamide) and improved stereochemical recognition at imprinted sites [29]. Moreover, Han et al. (2024) [30] constructed a novel Co–N–C electrocatalyst with atomically dispersed Co sites for CA15-3 detection, achieving signal amplification through Co–N active centers and ascorbic acid-mediated coupling of enzymatic and electrochemical processes.

**Figure 2:**
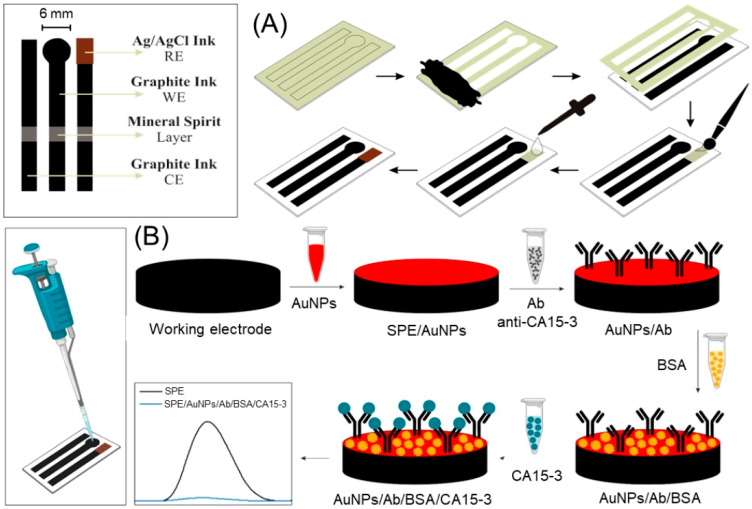
(**A**) Fabrication and (**B**) modification of SPE with AuNPs, anti-CA 15-3, and BSA to develop an immunosensor for CA 15-3 determination. Reproduce from reference Oliveira et al. [32] under the terms and conditions of the Creative Commons Attribution (CC BY) license.

It is important to highlight some dual sensing performance, being the case of Srilikhit et al. [31] approach, where a fluidic dual carbon electrode (Flu-iDCE) platform was developed via stencil printing and laser cutting for the simultaneous detection of Carcinoembryonic antigen (CEA) and CA125. The AuNP-modified DCE exhibited enhanced electrochemical performance, with a higher active surface area and roughness compared to conventional DCE. Applied to human serum samples, the label-free immunosensor showed strong potential for point-of-care cancer screening [31]. Oliveira et al. [32] demonstrate the potential of paper-based printed electrochemical sensors for CA15-3 determination in biological fluids (Figure 2). The proposed voltammetric immunosensor, integrating AuNPs and anti-CA 15-3, achieved high sensitivity (0.012 μA/U mL^−1^), low LOD (0.56 U mL^−1^), and robust specificity, although with a 1 h response time. The approach highlights the advantages of printed sensors, low cost, real-time analysis, and easy miniaturization, making them promising alternatives for monitoring breast cancer biomarkers [33].

Finally, a pure, dense, and adhesive layer of conductive CuNSs on Whatman^®^ (Maidstone, UK) filter paper using ion beam sputtering was developed by Naghib and colleagues [34]. The signal amplification was achieved through enzyme-mimicking catalysis, with Cu-PDA nanoparticles serving as both an antibody immobilization interface and an artificial enzyme for HQ oxidation. The proposed paper-based sensor exhibited a low LOQ (1.3 mU mL^−1^) and wide DLR (5–280 U mL^−1^), covering both healthy and patient ranges. Owing to the low cost, flexibility, and biocompatibility of paper, the device shows strong potential for CA15-3 point-of-care detection. However, being a signal-off system, it is more prone to noise compared to signal-on sensors, which typically offer higher sensitivity and lower LODs.

**Table 1:** Electrochemical biosensors for CA determination.

Nanomaterial	Biosensor Type	Linear Range	Detection Limit	Biomarker	Sample	Ref.
SPCE/PTBDES/AuNPs	Immunosensor	5–100 pg mL^−1^	1.20 pg mL^−1^	CA125	Serum	[21]
ONOH-3	Immunosensor	0.5–10 and 10–300 ng mL^−1^	0.805 μU mL^−1^	CA125	Serum	[25]
rGO-TEPA/ZIF-67@ZIF-8/Au	Immunosensor	0.001–1000 U mL^−1^	1.8 × 10^−5^ U mL^−1^	CA72-4	Serum	[26]
Ti_3_C_2_Tx/NH_2_-CNT	Immunosensor	1–500 U mL^−1^	1 μU mL^−1^	CA125	Serum	[22]
3DrGO/MWCNTs-Thi)UiO-66-NH_2_/ferrocenecarboxylic	Immunosensor	0.01–80 U mL^−1^	0.089 U mL^−1^	CA125	Serum	[27]
PAAm-co-PMBAm@MIP@3-MPA@Au-SPE	Immunosensor	0.01–100 U mL^−1^	0.001 U mL^−1^	CA15-3	Serum	[29]
ITO-Mn/ZnO-Au	Immunosensor	0.002–3 ng mL^−1^	0.5 pg mL^−1^	CA125	Serum	[23]
NiFe_2_O_4_NPs	Immunosensor	8.5–70 U mL^−1^	8.5 U mL^−1^	CA125	Serum	[24]
FluiDCE	Immunosensor	2–50 ng mL^−1^	0.6 U mL^−1^	CEA, CA125	Serum	[31]
Paper-based electrodes (f-SPE)	Immunosensor	2–16 U mL^−1^	0.56 U mL^−1^	CA15-3	Serum Sputum	[32]
CNTs	Immunosensor	0–300 U mL^−1^	0.005 ng mL^−1^	CA15-3	Serum	[33]
Ab/HQ/Cu-PDA/CuNSs/FP	Immunosensor	5–280 U mL^−1^	1.3 mU mL^−1^	CA15-3	Serum	[34]
NGO-PtCo	Aptasensor	0.05–200 U mL^−1^	4.1 × 10^−2^ U mL^−1^	CA15-3	Serum	[28]

Note: PTBDES/AuNPs: (PTB) (poly toluidine blue in deep eutectic solvent)/gold nanoparticles (AuNP); ONOH-3: Onion oil-based organic–inorganic hybrids; rGO-TEPA/ZIF-67@ZIF-8/Au: Zeolitic Imidazol Frameworks-67 doped with rGO-TEPA= reduced graphene oxide-tetraethylenepentamine anchored in AuPdRu trimetallic nanoparticles; Ti_3_C_2_Tx/NH_2_-CNT-SPCE: Ti3C2Tx-MXene/amino-functionalized carbon nanotube (NH2-CNT) modified screen-printed carbon electrode (SPCE); ITO-Mn/ZnO-Au: Mn-doped ZnO nanorod anchored in ITO substrate; NiFe_2_O_4_-NPs: NiFe2O4 magnetic nanoparticles; 3D-rGO/MWCNTs-Thi)UiO-66-NH2/ferrocenecarboxylic: UiO-66-NH2/ferrocenecarboxylic acid (UiO-66-Fc) doped with reduced graphene oxide/multi-walled carbon nanotube carboxylic acid-thionine; NGO-PtCo: nitrogen-doped graphene oxide decorated platinum cobalt nanoparticles; PAAm-co-PMBAm@MIP@3-MPA@Au-SPE: electrode based on gold surface (Au-SPE/3-MPA) doped with a copolymer of acrylamide and N,N′-methylenebisacrylamide (PAAm-co-PMBAm); CNTs: carbon-nanotubes; f-SPE: Screen-printed paper-based electrodes (f-SPE); FluiDCE: fluidic dual carbon electrode; Ab/HQ/Cu-PDA/CuNSs/FP: device based on cellulosic filter paper (FP) coated with Cu nanosponges (CuNSs) and then covered with Cu-doped polydopamine nanospheres (Cu-PDA) and grafted with hydroquinone (HQ).

### 3.2. Carcinoembryonic Antigen (CEA)

The development of highly sensitive CEA electrochemical biosensors relies on the strategic incorporation of nanomaterials. The most frequently used compounds are reduced graphene oxide (rGO), carbon nanotubes (CNTs), and AuNPs, often combined with metal oxides (e.g., MoO_3_, ZnO) to form nanocomposites. This synergistic combination increases the electrode’s active surface area, accelerates electron transfer, and enhances stability, overcoming the limitations of conventional biosensors. Furthermore, innovative platforms like MXenes, low-cost carbon dots, and MOF/COF hybrids are being explored to expand detection versatility.

In the pursuit of maximum sensitivity, detection strategies focus on EIS, which facilitates label-free detection by monitoring surface changes. Although not explicitly named in every instance, the high sensitivity achieved by electrochemical immunosensors and aptasensors implies the use of pulse techniques like DPV or SWV. An emerging trend is the integration of dual-signal output platforms (electrochemical and colorimetric) or the use of aptasensors to improve reliability, stability, and the capacity for internal result verification.

The analytical performance of these systems is validated by achieving ultra-low LODs, which is fundamental for clinical relevance as it enables the early detection of minimal biomarker concentrations. A crucial step in the research is the rigorous validation in real human serum samples, which confirms that the sensor’s performance is robust against the complexity of the biological matrix. These advancements demonstrate the strong potential of electrochemical biosensors for integration into Point-of-Care Testing (POCT) devices, even through integration with technologies like smartphones.

Some studies have focused on developing highly sensitive electrochemical immunosensors for CEA detection. For instance, Cotchim et al. [35] introduced a novel label-free immunosensor using a nanocomposite of gold nanoparticles, molybdenum trioxide, and chitosan on a porous graphene-modified screen-printed electrode [35]. This dual-electrode sensor was designed for the simultaneous detection of both CEA and carbohydrate antigen 19-9 (CA19-9), a key biomarker for cholangiocarcinoma. The sensor demonstrated excellent performance with a low detection limit and was validated using real human serum samples. In another study, Ranjan et al. [36] developed a high-throughput electrochemical immunosensor utilizing an ionic liquid-functionalized molybdenum trioxide-reduced graphene oxide (MoO3-rGO-IL) nanocomposite. This sensor achieved a remarkably low detection limit for CEA and was also effective in analyzing real serum samples [36]. Similarly, Shamsazar et al. [37] fabricated an immunosensor by modifying a glassy carbon electrode with an iron oxide-multi-walled carbon nanotube composite, leading to an ultra-low detection limit and high stability. In a different approach, Mehta et al. [38] developed a flexible, label-free immunosensor for CEA using nitrogen-rich mesoporous carbon as the substrate, which eliminated the need for coupling agents and simplified the fabrication process [38].

**Figure 3:**
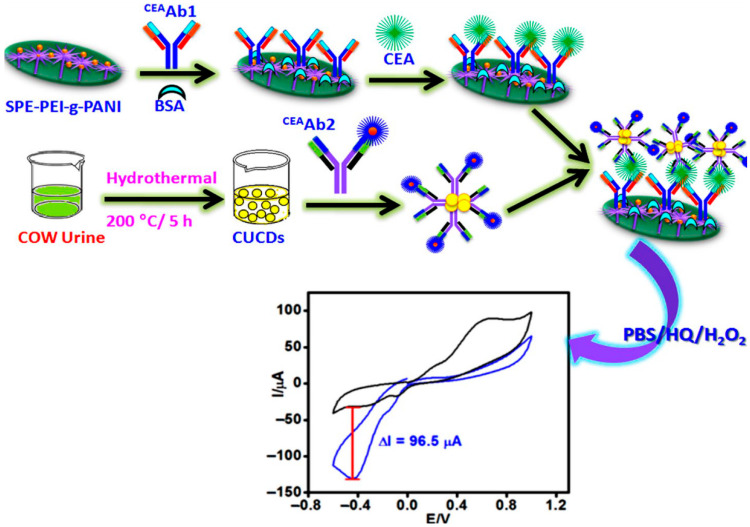
Construction of a sandwich immunosensor for CEA detection and signal amplification. Reproduce from reference Chellachamy Anbalagan et al. [39] under the terms and conditions of the Creative Commons Attribution (CC BY) license.

Innovative material synthesis and design have also played a crucial role. For example, Chellachamy Anbalagan et al. [39] created a cost-effective immunosensor using carbon dots synthesized from cow urine, which were bio-functionalized with an HRP-conjugated CEA antibody (Figure 3). This approach not only utilized animal waste but also resulted in a sensor with significantly higher sensitivity [39]. Meanwhile, Janduang et al. [40] synthesized flower-like zinc oxide nanoparticles. They combined them with graphene nanoplatelets to create a label-free immunosensor that could be integrated with a smartphone via NFC for POCT applications [40]. In a similar vein, Lei et al. [41] developed a homogeneous electrochemical sensor using carbon nanotube-bridged MXene electrode arrays integrated with a magnetic-bead-based immunoassay, which provided an ultra-low background signal for the highly sensitive detection of CEA.

Several studies have explored aptasensors, which use aptamers instead of antibodies for biomarker recognition, often providing advantages in stability and cost. Erkal-Aytemur et al. [7] presented a quartz crystal microbalance (QCM)-based aptasensor for CEA, demonstrating high selectivity and sensitivity suitable for clinical applications [7]. Rana et al. [42] introduced a hybrid of zirconia-gold nanoparticles (ZrO_2_-AuNPs) to enhance both the sensitivity and stability of their aptasensor for CEA detection, achieving a very low detection threshold and high diagnostic accuracy [42]. Yunussova et al. [43] developed a rapid, label-free aptasensor based on electrochemical impedance spectroscopy (EIS) for CEA, highlighting its potential for quick cancer screening. Furthermore, Shi et al. [19] presented a smartphone-based electrochemical aptasensing platform with a unique dual-signal output strategy to minimize false positives, while Li et al. [44] utilized a hybrid structure of metal–organic frameworks and covalent organic frameworks to create an aptasensor with a remarkably low detection limit.

**Figure 4:**
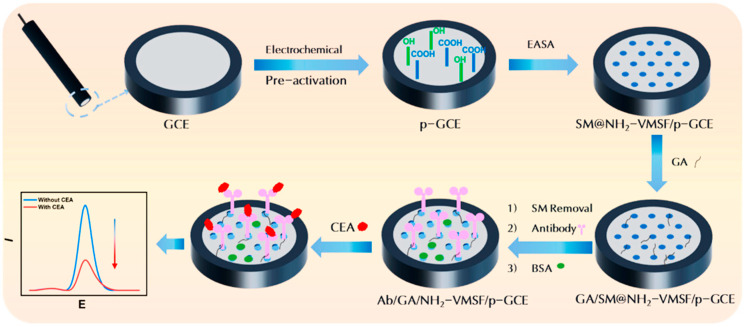
Schematic illustration of the construction of the immunosensor and the electrochemical detection of CEA. Reproduced from reference Zhou et al. [46] under the terms and conditions of the Creative Commons Attribution (CC BY) license.

Finally, some researchers have focused on creating multi-signal or integrated platforms to enhance reliability and user-friendliness. Zhang et al. [45] detailed a dual-mode homogeneous biosensor for CEA that provided both electrochemical and colorimetric signals on a microfluidic paper-based analysis device (μPAD), with an accompanying smartphone app for signal extraction. This dual-signal approach offers inherent self-verification, leading to more reliable results [45]. Zhou et al. [46] also developed a sensitive electrochemical immunosensor by integrating a biofunctionalized mesoporous silica nanochannel film with a carbon electrode, which effectively blocked the migration of a redox probe in the presence of CEA, leading to a measurable signal change (Figure 4). Table 2 summarizes the electrochemical biosensors for CEA determination.

### 3.3. Prostate-Specific Antigen (PSA)

The accurate detection of PSA, a vital biomarker for the early diagnosis of prostate cancer, has been transformed by integrating nanomaterials into electrochemical biosensors. The most frequently used materials are AuNPs, employed for both amplification and functionalization, and various carbon-based compounds, including carbon dots (CDs) and reduced graphene oxide (rGO). Innovative approaches like functionalized nanopores with Molecularly Imprinted Polymers (MIPs) and MOFs combined with peptides are also being explored. These nanomaterials work synergistically, increasing the surface area, enhancing electron transfer, and strengthening the stability of the platforms.

The highest sensitivity in these biosensors is primarily achieved through DPV. This technique offers ultra-low LODs by optimizing the signal-to-noise ratio. EIS is fundamental for label-free detection, efficiently monitoring surface changes. A crucial trend is the development of dual-mode systems, which combine electrochemical detection with complementary techniques like Surface-Enhanced Raman Spectroscopy (SERS) or optical detection, ensuring greater reliability and enabling internal verification of results.

The effectiveness of these biosensors is confirmed by achieving extraordinarily low LODs, many in the femtogram per milliliter (fg mL^−1^) range. These detection values far surpass the clinical requirements for PSA diagnosis and monitoring (>4 ng mL^−1^), enabling potentially much earlier detection. The analytical performance, which includes excellent selectivity, stability, and a broad linear range, is rigorously validated in real human serum samples. This validation in complex biological matrices underscores the potential of these platforms as reliable, high-performance tools for Point-of-Care Testing (POCT) in prostate cancer diagnosis.

A combination of electrochemical (EC) and surface-enhanced Raman spectroscopy (SERS) techniques was presented by Yaiwong et al. [47] as a dual-mode immunosensor for PSA detection. The device was fabricated by modifying a screen-printed carbon electrode (SPCE) with a nanocomposite consisting of gold nanoparticles (AuNPs) deposited onto two-dimensional molybdenum disulfide (2D-MoS_2_). A primary antibody (Ab1) was immobilized on the modified electrode for the specific detection of the target PSA. Dual-signaling nanotags (TMB/Ab2/AuNPs) were then prepared by conjugating AuNPs with a secondary antibody (Ab2) and a probe molecule (TMB), which exhibit strong responses for both SERS and EC. The formation of a sandwich-type immunocomplex (Ab1-PSA-Ab2) in the presence of PSA enables detection with both techniques. The developed system demonstrated excellent selectivity and sensitivity, with low LODs of 3.58 pg mL^−1^ for the EC mode and 4.83 pg mL^−1^ for the SERS mode, suggesting a high detection capability. In conclusion, the high sensitivity and efficacy of the sensor in analyzing biological samples make it a promising tool for the early diagnosis of cancer.

Uruc et al. [48] described a new label-free immunosensor with promising applicability for clinical prostate cancer screening and diagnosis. The sensing interface was constructed from a hybrid film of 3,4-ethylenedioxythiophene (EDOT), 3-methylthiophene (3MT) and AuNPs, enabling highly sensitive PSA determination. Ferricyanide/ferrocyanide ([Fe(CN)_6_]^3−^/^4−^) was used as the redox probe, and differential pulse voltammetry (DPV) was employed to correlate the electrochemical signal with PSA concentration. The analytical response was linear from 0.1 to 5.0 × 10^4^ pg mL^−1^, with a detection limit of 0.083 pg mL^−1^. Owing to its low cost, good reproducibility, selectivity and accuracy, this immunoplatform is suitable for adaptation into point-of-care diagnostic devices. Moreover, Chellachamy Anbalagan et al. [49] developed a novel electrochemical biosensor for the detection of PSA using a sensing platform based on carbon dots functionalized polyaniline (CDs@PANI) [49]. The sensor was constructed by covalently grafting biomass-derived CDs onto a PANI surface to form a biocompatible and fluorescent nanocomposite, CDs@PANI. This composite served as an immobilization matrix on a screen-printed carbon electrode (SPE). The detection mechanism was a sandwich electrochemical immunoassay, where a capture antibody (PSAAb1) was immobilized on the CDs@PANI platform. Following this, the PSA antigen and a horseradish peroxidase (HRP) conjugated detection antibody (PSAAb2) were sequentially introduced to form an immunocomplex. This sensor showed a broad linear detection range of 0.01–60 ng mL^−1^ and a low detection limit of 20 pg mL^−1^. The developed CDs@PANI-based biosensor showed superior analytical performance, including good sensitivity, specificity, stability, and reproducibility for PSA detection in real human serum samples.

In other work, Wang et al. [50] presented a novel electrochemical sensing strategy for label-free dual-biomarker detection, utilizing a chronopotentiometric nanopore sensor modified with stimulus-responsive molecularly imprinted polymers (MIPs). The sensor’s construction was based on a combination of nanopores and a polymeric membrane chronopotentiometric sensor. The solid-state nanopores, made from anodic aluminum oxide (AAO), were functionalized with two different stimulus-responsive MIPs to recognize two distinct biomarkers selectively. For detecting alpha-fetoprotein (AFP), a pH-sensitive MIP was created using 3-aminobenzeneboronic acid (APBA) as the functional monomer. For PSA detection, a temperature-sensitive MIP was synthesized using an N-isopropylacrylamide (NIPAAm) functionalized aptamer. The sensor demonstrated high sensitivity with LOD of 0.17 ng mL^−1^ for AFP and 0.42 ng mL^−1^ for PSA. The research demonstrates a significant step toward improving early disease diagnosis, particularly for cancers like hepatocellular carcinoma and prostate cancer, by allowing for the simultaneous detection of multiple biomarkers from a single sample.

Redondo-Fernández et al. [51] reported an analytical, low-cost, smartphone-based system for the ultrasensitive detection of PSA. In this case, the system offers two detection alternatives: an electrochemical method using a portable potentiostat, and an optical method that uses a smartphone coupled with magnifying lenses. The sensor platform was an indium tin oxide-coated polyethylene terephthalate (ITO-PET) platform, which serves as a solid support for the immunoassay. The detection mechanism is a sandwich-type immunoassay where a specific anti-PSA antibody is immobilized on the ITO-PET surface. A second, anti-PSA antibody, tagged with AuNPs, is then used for the recognition step. A key step for signal amplification is a controlled silver electrodeposition on the surface of the AuNPs, which significantly enhances their size from the nano to the micrometer scale. The proposed strategies exhibited very low LOD of 102 fg mL^−1^ for electrochemical detection and 37 fg mL^−1^ for optical detection. This immunosensor also showed excellent selectivity and performance, capable of quantifying biomarkers at clinically relevant values without any pretreatment of the sample.

Ren et al. [52] showed an electrochemical peptide biosensor, 2FcP-GA-GDY(Fe)@NMIL-B, developed for the highly selective, ultrasensitive, and ultrastable detection of PSA. The sensor’s construction was based on a dual chemical bonding strategy to enhance both electron transport and peptide immobilization. The first chemical bond is a C-Fe-O interface created by incorporating high-conductivity Fe-Graphdiyne (Fe-GDY) into a metal–organic framework (MOF) material, NH_2_-MIL88B(Fe) (NMIL88B). This bond significantly accelerates electron transport. The second chemical bond is a Schiff-base (-N=C-) formed with glutaraldehyde (GA) as a crosslinking agent, which firmly links a ferrocene-labeled peptide (2FcP) to the electrode carrier at high density. When PSA is introduced, it cleaves the specific peptide, releasing ferrocene and leading to a decrease in the electrical signal, which enables sensitive detection. The study reports an ultra-low LOD of only 0.94 fg mL^−1^. The sensor also showed an extended linear response range from 10 fg mL^−1^ to 50 ng mL^−1^. In conclusion, the 2FcP-GA-GDY(Fe)@NMIL-B sensor demonstrates superior analytical performance, including excellent selectivity and stability in human serum samples, making it a promising tool for clinical use in the early diagnosis of prostate cancer.

Also, Rahman et al. (2024) developed a novel, label-free impedimetric biosensor for the precise detection of tumor-associated biomarkers for prostate cancer [53]. This research used Maackia amurensis (MAA) lectin as a recognition element to identify cancer-associated aberrant glycosylation of PSA. The sensor was constructed by immobilizing MAA lectin onto gold-interdigitated microelectrodes, achieved through a series of surface modifications that included the formation of a self-assembled monolayer using 11-mercaptoundecanoic acid (MUA) and the activation of carboxylic acid groups via a crosslinking agent for lectin immobilization. The sensing mechanism was based on measuring the impedance response, which changes when the MAA lectin binds to the PSA-containing glycans. The sensor showed a LOD of 3.574 pg mL^−1^ and a concentration range of 0.01–100 ng mL^−1^. A crucial finding was that the MAA lectin preferentially recognizes α2,3-linked sialic acid in serum PSA, which is a specific glycan known to be a valuable biomarker for improving the specificity of prostate cancer diagnoses. This biosensor offers a miniaturized and cost-effective platform for detecting the PSA biomarker.

Another study described an aptamer-based electrochemical biosensor for prostate cancer diagnosis that employed a novel two-dimensional (2D): 2D composite of reduced graphene oxide (rGO) and graphitic carbon nitride (g-C_3_N_4_), further decorated with AuNPs [54]. This sensor was constructed by modifying a glassy carbon electrode (GCE) with the rGO/g-C_3_N_4_/AuNPs composite, upon which aptamer chains were immobilized. This device exhibited remarkable analytical performance, showing high selectivity toward PSA when compared to potential interfering substances like bovine serum albumin (BSA) and glucose. Under optimized conditions, the sensor achieved a rapid detection time of 30 min and a low LOD of 0.44 fM (femtomolar). The present method showed a significant advancement in early prostate cancer diagnosis with its validation using real serum samples, demonstrating its potential for clinical use.

On the other hand, Yue et al. [55] published an electrochemical biosensor for the ultrasensitive detection of PSA in human serum, using a platform based on α-Fe_2_O_3_/Fe_3_O_4_@Au nanocomposites and magnetically induced self-assembly (MISA) technology. This sensor was constructed in two main steps. First, magnetic α-Fe_2_O_3_/Fe_3_O_4_ nanoparticles were synthesized, and then α-Fe_2_O_3_/Fe_3_O_4_@Au nanocomposites were prepared by reducing HAuCl_4_ with NaBH_4._ The biosensor was assembled by immobilizing a sulfhydryl-modified PSA aptamer onto the material surface via Au–S bonds, followed by the self-assembly of magnetic nanoparticles onto a magnetic glassy carbon electrode (MGCE) through MISA technology. The developed biosensor demonstrated excellent analytical performance, with a linear response range from 100 fg mL^−1^ to 100 ng mL^−1^ and a low LOD of 0.78 pg mL^−1^. This biosensor offered a valuable tool for clinical PSA detection, particularly for point-of-care testing (POCT). The present study confirms the sensor’s excellent repeatability, stability, selectivity, and reproducibility, and its capability to analyze real samples, laying a strong foundation for future practical applications.

**Figure 5:**
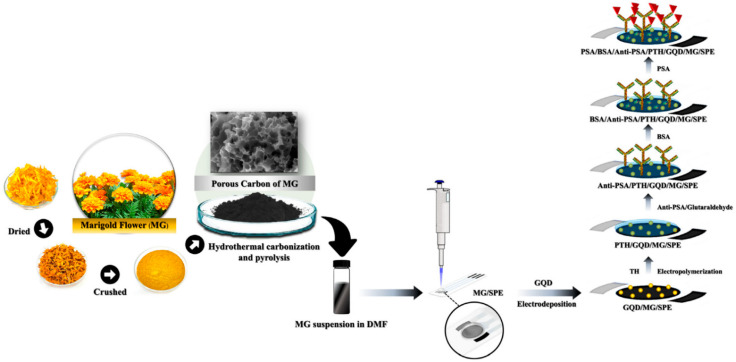
Fabrication process of the proposed label-free electrochemical immunosensor. Reproduce from reference Cotchim et al. [56] under the terms and conditions of the Creative Commons Attribution (CC BY) license.

Moreover, Cotchim et al. [56] reported an electrochemical immunosensor for the detection of PSA, utilizing activated carbon from marigold flowers (MG) as a key component. The sensor was meticulously constructed using a series of modifications (Figure 5). First, activated carbon from marigold flowers (MG) was synthesized through hydrothermal carbonization and pyrolysis. This material was then modified with graphene quantum dots, which significantly enhanced electron transfer. Poly(thionine) (PTH) was subsequently grafted onto this modified surface, and the amine groups of PTH were used to bond with anti-prostate-specific antigen (Anti-PSA) via glutaraldehyde. This intricate layer-by-layer assembly resulted in a sensor with an improved electron transfer layer and a high affinity for the target biomarker. It exhibited two distinct linear detection ranges: 0.0125 to 1.0 ng mL^−1^ and 1.0 to 80.0 ng mL^−1^. The sensor’s high sensitivity was evidenced by its low LOD of 0.005 ng mL^−1^ and a quantification limit of 0.017 ng mL^−1^. In conclusion, this research successfully developed a highly sensitive and reliable unlabeled electrochemical immunosensor for PSA detection in serum samples. Table 3 summarizes electrochemical biosensors for PSA determination.

### 3.4. miRNA, DNA, EVs and Cells

Electrochemical biosensors are rapidly emerging as a powerful tool in cancer diagnostics, offering sensitive, specific, and often portable platforms for detecting a range of crucial biomarkers, including microRNAs (miRNAs), exosomes, and tumor cells. These innovative approaches aim to overcome the limitations of traditional diagnostic methods, such as their complexity, high cost, and lengthy analysis times. Recent research highlights significant progress in this field, focusing on diverse biomarkers, often integrating novel nanomaterials and amplification strategies to achieve superior analytical performance [57,58,59].

On the other hand, DNA probes, aptamers, and PNA (peptide nucleic acid) probes are widely used in miRNAs biosensors, each with distinct advantages and limitations. DNA probes are chemically robust, easy to synthesize, and cost-effective, but their negatively charged backbone can reduce sensitivity due to electrostatic repulsion with target miRNAs. Aptamers offer exceptional specificity and binding affinity with the ability to undergo chemical modifications for enhanced sensitivity and selectivity; however, RNA aptamers are prone to degradation by nucleases, which limits their stability in biological samples. PNA probes, with a neutral backbone, provide higher stability against nucleases and stronger affinity for miRNAs, improving sensitivity and shelf-life, but their lower solubility in aqueous media requires careful probe design. Overall, these probes contribute significantly to miRNAs detection in biosensors, with trade-offs between stability, sensitivity, cost, and ease of use influencing their application choice [58,59,60].

#### 3.4.1. miRNA Detection: A Focus on Early Cancer Biomarkers

MicroRNAs (miRNAs) are small, non-coding RNAs that play critical roles in post-transcriptional gene regulation and have emerged as valuable biomarkers for the early diagnosis and prognosis of various cancers. Their low abundance, short sequence length, and high sequence homology, however, make their detection particularly challenging. Recent advances in electrochemical biosensors, combined with nanomaterials such as gold nanoparticles, carbon-based structures, and metal oxides, have enabled highly sensitive and selective detection of miRNAs in biological samples.

Table 4 summarizes representative electrochemical strategies for miRNA detection, highlighting the diversity of nanomaterials employed, the type of biorecognition element (DNA probe, aptamer, or peptide nucleic acid), analytical performance, and cancer-related applications.

miRNA detection is a significant area of research due to their role as critical cancer biomarkers. Several recent studies highlight the development of highly sensitive electrochemical aptasensors and biosensors for miRNA detection. For instance, a novel dual-targeted electrochemical aptasensor utilizes gold nanorods (AuNRs) and aptamer-functionalized gold nanoparticles (AuNPs) on screen-printed carbon electrodes (SPCEs) to simultaneously detect neuroblastoma-associated miRNA-181 and miRNA-184 with remarkable LODs of 5.10 aM and 9.39 aM, respectively [57]. This amplification-free aptasensor offers a user-friendly and easily engineered platform for early cancer diagnosis and screening.

Another innovative electrochemical biosensor focuses on microRNA-21 (miRNA-21). This biosensor integrates peptide nucleic acid (PNA)-DNA hetero-three-way junction (H3WJ) formation with enzyme-free target-recycling catalytic hairpin assembly (CHA) amplification, achieving an exceptionally low detection limit of 0.15 fM for miRNA-21 [58]. The PNA-DNA H3WJ structure enhances stability and sensitivity, and the biosensor demonstrated excellent reproducibility and long-term stability, proving its utility in human cancer cells. Similarly, aluminum-doped zinc oxide (AZO) nanostars have been employed as a novel electrode modification material to enhance the conductivity and surface area of an electrochemical biosensor for highly sensitive miRNA-21 detection [59]. The unique morphology and high electron transfer efficiency of AZO nanostars contribute to a remarkably low detection limit and a wide linear range for miRNA-21 quantification [60].

**Figure 6:**
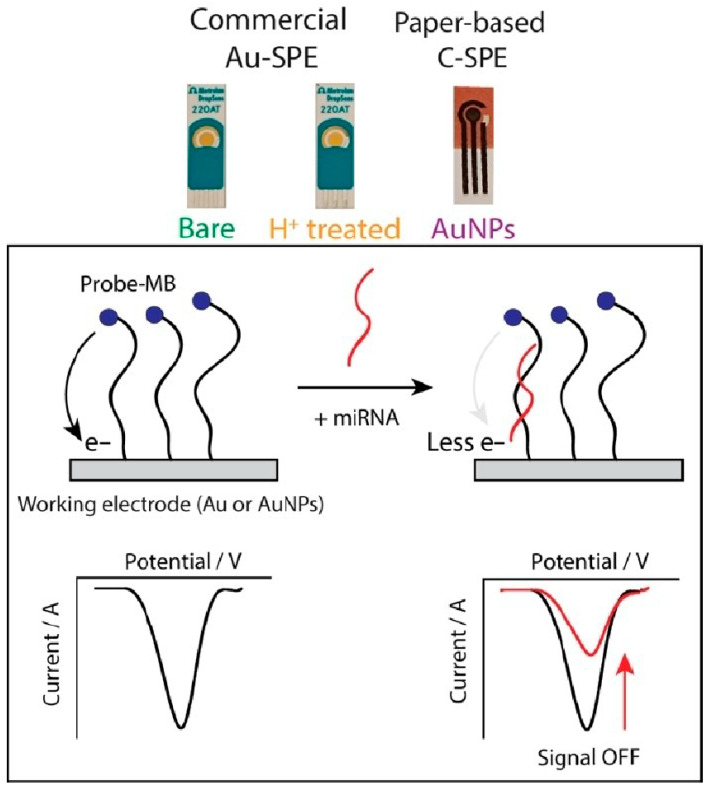
Schematic representation of the screen-printed electrodes (SPEs) evaluated and the signal-OFF electrochemical sensing strategy for miRNA. Top: commercial bare Au-SPE, acid-treated commercial Au-SPE, and AuNP-modified paper-based carbon SPE. Bottom: methylene blue–labeled DNA probes immobilized on Au or AuNP working electrodes undergo hybridization with the target miRNA, which restricts electron transfer and produces a progressive decrease in current as the miRNA concentration rises. Figure adapted from Raucci et al. [62], distributed under the Creative Commons Attribution (CC BY) license.

Further advancements in miRNA detection include a CRISPR-empowered electrochemical biosensor, PER-E-CRISPR, for sensitive and target amplification-free detection of miRNA-21 [61]. This biosensor leverages CRISPR/Cas13a and primer exchange reaction (PER) for dual-signal amplification, achieving a low LOD of 30.2 fM without the need for pre-amplification. The practicality of SPEs for miRNA detection in liquid biopsy, particularly for lung cancer management, has also been explored, demonstrating their potential for decentralized and user-friendly diagnostic solutions using both commercial and hand-made SPEs (Figure 6) [62].

For non-small cell lung cancer (NSCLC), an electrochemical biosensor employing a gold/multiwall carbon nanotube (Au/MWCNT) nanocomposite-based sandwich platform has been developed for the highly sensitive detection of microRNA-223 (miR-223) [63]. This platform offers enhanced sensitivity and selectivity without complex sample preparation. Moreover, a paper-based strategy has been introduced to enhance the sensitivity of electrochemical miRNA detection, focusing on miR-224, a lung cancer biomarker [64]. This approach utilizes an external chromatographic wax-patterned paper-based disk to preconcentrate the sample, significantly lowering the detection limit and offering a more affordable and sustainable method for cancer biomarker detection.

**Figure 7:**
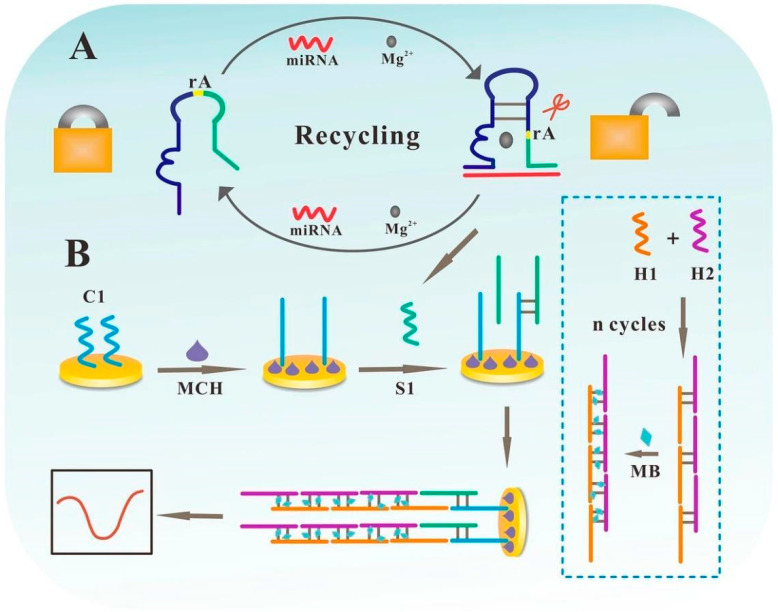
Schematic illustration of the electrochemical biosensing strategy for extracellular vesicle miRNA detection. (**A**) The target miRNA participates in a cyclic reaction with NAL, repeatedly generating additional nucleic acid strands. (**B**) These newly produced strands are recognized and assembled on the electrode surface, producing an amplified electrochemical signal. Figure adapted from Zhu et al. [65], distributed under the Creative Commons Attribution (CC BY) license.

Another notable development for lung adenocarcinoma (LUAD) diagnosis involves an electrochemical biosensor for fucosylated extracellular vesicles (EVs) containing miR-4732-5p [65]. This biosensor employs a dual amplification strategy combining a Mg^2+^-dependent DNAzyme splitting nucleic acid lock (NAL) cycle and hybridization chain reaction (HCR) to achieve high sensitivity (Figure 7).

Further progress in miRNA sensing has led to the development of a paper-based electrochemical device for the detection of miRNA-652, a biomarker linked to triple-negative breast cancer (TNBC) [66,67,68,69]. This device, featuring an AuNP-modified office paper-based screen-printed electrode, demonstrated satisfactory repeatability and selectivity, with an additional 10-fold improvement in detection limit achieved through pre-concentration. Addressing the detection of other crucial miRNAs, Dulgerbaki and Oksuz [66] presented a label-free electrochemical biosensor for miRNA-21 utilizing a glassy carbon electrode modified with a nanocomposite of iron oxide nanoparticles and PEDOT: PSS, exhibiting high sensitivity and selectivity. Expanding on amplification methods, Yu et al. [68] designed an electrochemical biosensor for miRNA-21 based on photoinduced electron/energy transfer reversible addition–fragmentation chain transfer (PET-RAFT) polymerization as a signal amplification strategy, reaching an ultralow detection limit of 12.4 aM. Additionally, an electrochemical biosensor based on an atomic layered composite of graphene oxide (GO) and graphene (G) has been developed for selective and sensitive miRNA-21 detection, offering a broad linear detection range and a low LOD of 3.18 fM [61].

In a novel approach to amplification-free detection, Ali et al. [70] engineered a biosensing platform for the ultrasensitive and enzyme-free electrochemical detection of hsa-miR-141 using PNA-functionalized Ti_3_C_2_Tx MXene nanosheets, demonstrating an impressive detection limit of 40 aM without the need for complex amplification steps or nanomaterial labels [70]. Furthermore, to enable comprehensive diagnosis, Zhou et al. [71] established a novel electrochemical sensor, PER-CRISPR/Cas9-E, for the sensitive and simultaneous detection of dual miRNAs (miRNA-21 and miRNA-155), integrating primer exchange reaction (PER) for signal amplification with the precise cleavage capabilities of the CRISPR/Cas9 system. Finally, Li et al. [67] developed a highly sensitive and specific electrochemical biosensor for miRNA-155 detection by integrating magnetic beads-assisted split DNAzyme cleavage with the assembly of functionalized covalent organic frameworks (COFs), achieving an ultralow detection limit of 1.2 fM through multiple signal amplification.

A significant stride in ultrasensitive detection of small extracellular vesicle (sEV)-derived miRNAs has been made with the development of a novel electrochemical biosensor utilizing a turbo-like localized catalytic hairpin assembly (T-CHA) strategy [72]. This biosensor employs a dual localization approach with gold nanoparticles (AuNPs) and sophisticated DNA nanotechnology, enabling robust signal amplification, with an impressive LOD of 5.24 aM and proven efficacy in real clinical serum samples. Addressing the urgent need for improved early diagnosis of ovarian cancer, a “silent killer” often detected at late stages, another electrochemical nanobiosensor platform has been developed for the simultaneous and sensitive detection of multiple miRNAs [73]. This biosensor leverages carboxylated graphene oxide (GO-COOH) modified SPCEs to immobilize specific DNA probes for miR-200c and miR-141. The use of DPV yields linear ranges of 0.1 pM–10 nM, with corresponding LODs of 0.029 pM and 0.026 pM.

**Figure 8:**
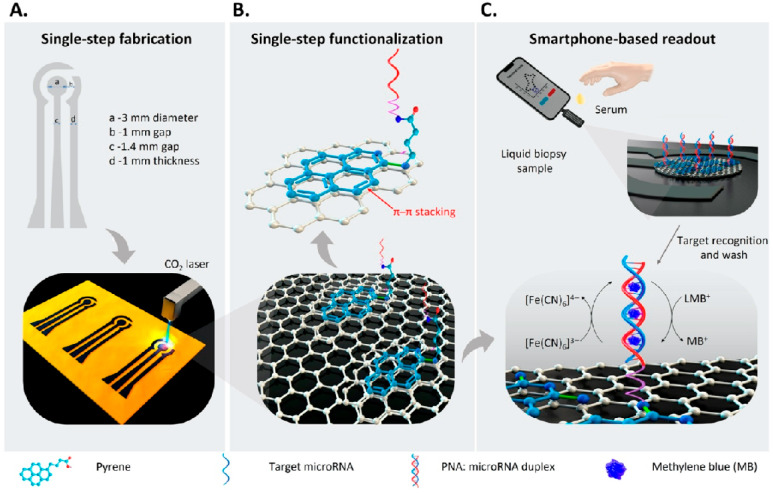
Illustration of the PNA-functionalized laser-induced graphene (LIG) biosensor. (**A**) The sensor is produced in a single step by laser scribing a polyimide (PI) film to generate the LIG electrode. (**B**) In a subsequent one-step process, pyrene-modified PNA probes are immobilized on the graphene surface through π–π interactions. (**C**) Target miRNA hybridization forms a PNA–miRNA duplex bearing methylene blue (MB), whose redox response is monitored electrochemically and read out via a smartphone-based system. Figure adapted from Barman et al. [74], distributed under the Creative Commons Attribution (CC BY) license.

Beyond enhanced sensitivity and multiplexing, efforts are also focused on creating portable and user-friendly biosensors for POCT settings. A groundbreaking smartphone-interfaced electrochemical biosensor has been introduced for the ultrasensitive, rapid, and robust detection of miRNA cancer biomarkers (Figure 8) [74]. This device addresses limitations of high costs and bulky equipment by using laser-induced graphene (LIG) as the sensing platform and a unique π-π stacking immobilization strategy for peptide nucleic acid (PNA) probes. Its single-step fabrication and functionalization, coupled with DPV detection using methylene blue as a redox indicator, achieved an ultra-low LOD of 48.3 aM and a broad dynamic range.

Further advancing the frontiers of miRNA detection, an innovative electrochemical biosensor for ultrasensitive detection of microRNA-182-5p (miRNA-182-5p) has been developed [75]. This biosensor employs a novel strategy based on the target-driven cascade amplified assembly of COFs on a tetrahedral DNA nanostructure (TDN). COFs serve as efficient nanocarriers for electroactive Prussian blue (PB), providing a simple and effective signal reporter. The core of its amplification strategy integrates a chain hybridization amplification (CHA) reaction with a manganese (II)-powered DNAzyme system, ensuring remarkable signal enhancement. The TDN’s multi-recognition domains also improve the capture efficiency of the PB-COFs, leading to an impressive detection limit of 2.5 fM for miRNA-182-5p.

#### 3.4.2. Extracellular Vesicles Detection: Non-Invasive Biomarkers

Exosomes are nanosized extracellular vesicles released by cells that transport proteins, lipids, and nucleic acids, thereby mirroring the physiological and pathological status of their cells of origin. Because tumor-derived exosomes are abundant and detectable in body fluids such as serum and urine, they represent promising non-invasive biomarkers for cancer diagnosis and monitoring [76,77,78]. Electrochemical biosensors, often coupled with nanomaterials and specific capture probes, have been developed to achieve sensitive and selective exosome detection. These platforms are particularly relevant since they address the limitations of conventional exosome assays (e.g., ultracentrifugation, which is time-consuming and requires specialized equipment) [79,80]. Table 5 presents selected examples that illustrate how nanomaterial-assisted biosensors can reduce assay time while maintaining clinical relevance.

Electrochemical biosensors are proving effective for the detection of exosomes, which are vital non-invasive biomarkers for cancer. A homogeneous and portable electrochemical sensing platform has been developed for the accurate detection of breast cancer exosomes [76]. This platform utilizes a DNAzyme-induced DNA walker and enzyme-catalyzed amplification strategies, eliminating the need for complex washing steps and tedious electrode modifications, making it suitable for point-of-care diagnostics [76].

The sensitive detection of small extracellular vesicles (EVs), also known as exosomes, has been achieved using a colloidal quantum dots (CQDs)-modified electrochemical sensor [77]. This sensor, modified with a CD63 antibody and utilizing PbS CQDs, demonstrates rapid and highly sensitive electrochemical responses with a remarkably low detection limit of 19 particles per mL for sEVs, indicating its potential for membrane-biomarker-based diagnostics. Furthermore, a dual-amplified electrochemical sensing platform has been developed for the sensitive and accurate detection of exosomal microRNA-21 (miR-21) for breast cancer diagnosis [78]. This platform integrates ternary hybridization-based recognition with a perchlorate-assisted electrocatalytic cycle, offering a highly sensitive and specific method without the need for pre-amplification.

Exosomes, as crucial biomarkers for cancer diagnosis, have been a focal point for the development of electrochemical immunosensors. Sahraei et al. [79] introduced a flexible, paper-based electrochemical immunosensor (Exo-sensing paper) for direct detection of exosomes in serum samples. This innovative system incorporates a 3D porous nanocomposite of nickel nanofoam with graphene oxide and gold nanoparticles, significantly enhancing the sensor’s surface area and conductivity for increased antibody loading. Similarly, Sazaklioglu et al. [80] developed a lab-on-paper-based immunosensor for label-free exosome detection using electrochemical impedance spectroscopy (EIS), offering a portable and disposable solution for early cancer or metastasis detection. This sensor utilizes gold particles on a carbon working electrode conjugated with anti-CD9 antibodies, and the binding of exosomes causes a measurable impedance change. Further pushing the boundaries of sensitivity, Zhang et al. [81] designed an electrochemical aptasensor for exosome detection based on a dual nucleic acid amplification strategy combining primer exchange reaction (PER) and rolling circle amplification (RCA). This innovative approach enables the detection of exosomes at remarkably low concentrations, showcasing the power of amplification techniques in overcoming the challenges of low biomarker abundance.

#### 3.4.3. Tumor Cell and Circulating Tumor DNA Detection: Early Cancer Screening

Circulating tumor cells (CTCs) and circulating tumor DNA (ctDNA) provide direct information about cancer progression and genetic alterations, offering opportunities for minimally invasive “liquid biopsies.” Electrochemical biosensors have been engineered to identify these biomarkers with high sensitivity and specificity, exploiting nanostructured electrodes and molecular recognition elements such as aptamers, antibodies, or DNA probes [82]. These systems allow for the detection of extremely low concentrations of tumor-derived material in clinical samples, which is crucial for early diagnosis and disease monitoring. Table 6 summarizes representative electrochemical biosensors for CTCs and ctDNA detection, detailing their analytical performance and potential applications in cancer diagnostics [83,84].

The detection of tumor cells themselves is another critical aspect of early cancer screening and diagnosis. Tang et al. [17] designed an integrated microfluidic cytosensor for the sensitive and accurate detection of hepatoma cells (HepG2 cells), a biomarker for hepatocellular carcinoma (HCC). This cytosensor employs a dual-site recognition and dual-mode signal readout strategy, combining electrochemical and colorimetric detection, providing a robust platform for circulating tumor cell (CTC) analysis in liquid biopsies. In addition, a new electrochemical approach for ultrasensitive detection of MUC1-expressing tumor cells has been proposed, based on a proximity labeling-assisted multiple signal amplification strategy [82]. This biosensor, which involves the formation of a hemin/G4-DNA complex to catalyze silver deposition, ensures high sensitivity in tumor cell detection, offering a promising tool for early cancer screening.

Yan et al. [83] introduced a highly sensitive cell-based electrochemical biosensor for hepatocellular carcinoma (HCC) detection, leveraging a single-chain variable fragment (scFv) engineered from a recombinant humanized monoclonal antibody. By immobilizing the His-tagged scFv onto a nickel-based nanomaterial-modified electrode, the biosensor achieved enhanced binding affinity and specificity for HCC cells [83]. The platform demonstrated a wide dynamic range (10^2^–10^7^ cells mL^−1^) and an exceptionally low detection limit of 2 cells mL^−1^, with rapid detection within 5 min. Its ability to selectively identify HCC cells even in complex biological matrices, such as mouse liver tissue extracts, underscores its potential for early-stage cancer diagnostics and highlights the growing utility of antibody fragment-based biosensors in liquid biopsy applications.

Beyond exosomes and miRNAs, electrochemical biosensors are also being developed for the detection of other critical cancer biomarkers, such as ctDNA and specific cancer cell lines.

**Figure 9:**
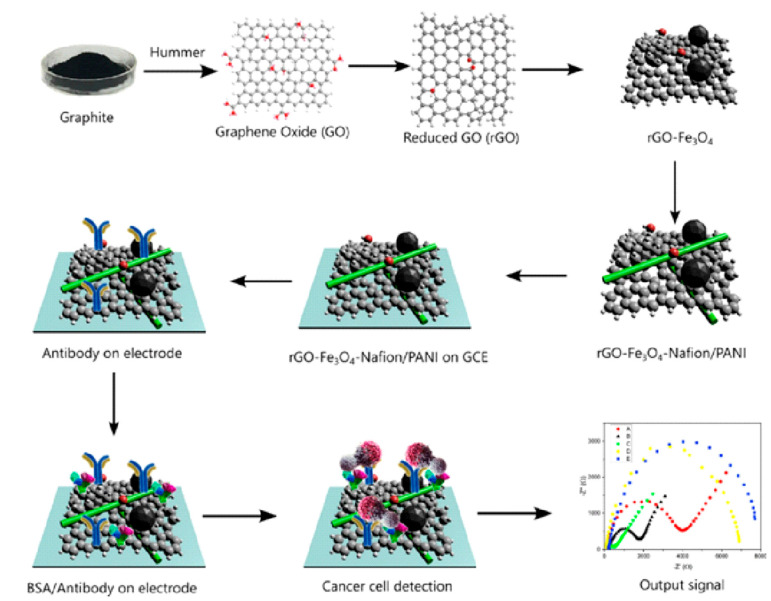
Schematic process diagram for the fabrication and functionalization of sandwich rGO/Fe_3_O_4_/Nafion/PANI for the detection of SK-BR3 cell line. Reproduce from reference Hosseine et al. [84] under the terms and conditions of the Creative Commons Attribution (CC BY) license.

Furthermore, for the detection of specific cancer cell lines, Hosseine et al. [84] developed a label-free electrochemical biosensor for the ultrasensitive detection of the SKBR3 cell line, a key indicator of HER2 breast cancer (Figure 9). This biosensor incorporates a sophisticated nanocomposite of green-synthesized reduced graphene oxide, iron oxide nanoparticles, Nafion, and polyaniline, achieving a remarkably low detection limit of 5 cells mL^−1^. Yang et al. [85] introduced a novel electrochemical paper-based analytical device (ePAD) for the ultrasensitive detection of ctDNA from mice serum, enhanced by the integration of MBene as a novel material and a magnetic clutch probe for efficient pre-enrichment of target ctDNA. This ePAD exhibited an exceptionally low detection limit of 178–216 fM [85]. For the direct and pre-enrichment-free detection of hepatocellular carcinoma (HCC)-specific ctDNA, Çağlayan Arslan et al. [86] developed a PDMS and MEMS-based microfluidic sensor, demonstrating promising limits of detection in various matrices, including human plasma and clinical samples.

The detection of ctDNA through liquid biopsy is also gaining traction for non-invasive cancer diagnosis and monitoring. A novel method for ultrasensitive and rapid detection of ctDNA utilizes surface-confined gene amplification on dispersible magnetic nano-electrodes [87]. This system integrates Fe_3_O_4_-Au core–shell nanoparticles directly into polymerase chain reactions (PCR), allowing these nanoparticles to function as “nano-electrodes” for in situ amplification and accumulation of target ctDNA. This technology achieves detection within one hour and boasts an exceptional ultra-low detection limit of ~3 aM, making it highly sensitive to even minute quantities of ctDNA. Its successful validation in serum samples from breast cancer patients underscores its promise for earlier and more accurate cancer diagnosis, recurrence monitoring, and assessment of therapeutic efficacy.

### 3.5. Other Relevant Epithelial Biomarkers

The following paragraphs highlight significant progress in developing electrochemical biosensors for detecting various cancer biomarkers. While each study focuses on a specific cancer type and biomarker, these studies involve novel nanomaterials, innovative sensor architectures, and cost-effective fabrication methods [88,89,90].

EGFR, a type of transmembrane glycoprotein, is frequently found to be overexpressed or to have an aberrant expression in several solid tumors, such as those in the lung, breast, and glioblastoma. Its physiological functions include regulating the development and homeostasis of epithelial tissues. However, it also plays a significant role in several pathological processes, including the proliferation of tumor cells, angiogenesis, invasion, metastasis, and the prevention of apoptosis. Consequently, EGFR’s characteristics make it a dependable diagnostic biomarker for cancers where it is involved, mainly lung and breast cancer [90,91].

Yue et al. [88] reported an electrochemical biosensing system for the sensitive detection of the EGFR biomarker. This system used magnetic nanocomposites and a magnetic-induced self-assembly (MISA) technique to enhance detection efficiency and enable point-of-care (POC) testing. The sensor was constructed using magnetic Fe_3_O_4_/α-Fe_2_O_3_@Au nanocomposites as both signal amplifiers and the immobilization matrix for specific aptamer probes (ssDNA-APT). This sensor demonstrated a wide linear detection range from 0.1 to 1000 ng mL^−1^ and a low LOD of 0.18 ng mL^−1^. The sensor’s ability to detect EGFR with high precision and its potential for real sample analysis demonstrate its value as a tool for early diagnosis and monitoring of EGFR-related cancers.

In other work, Zhang et al. [89] reported a bimodal biosensor for the detection of the EGFR L858R mutation, a key biomarker for specific non-small cell lung cancer (NSCLC) patients. The sensing platform relies on a dual amplification strategy. In the presence of the EGFR L858R mutation, the target sequence is not cleaved by the Mscl restriction enzyme, which in turn activates the first CRISPR–Cas12a system. This activation prevents nanomagnetic beads from capturing the fluorescein-labeled hybridization chain reaction (HCR) products, resulting in a fluorescence “signal-off” output. At the same time, the cleavage activity of a second CRISPR–Cas12a module is suppressed, allowing nanomaterial-based labels on the electrode to retain a strong electrochemical response, thus generating a “signal-on” electrochemical signal. This biosensor demonstrated exceptional analytical performance, with a dynamic detection range spanning from 10 fM to 1 µM and a LOD of 372 aM. This system also exhibits excellent specificity, reproducibility, stability, and recovery rates, representing a grand promise for early disease diagnosis and personalized therapeutic strategies for NSCLC patients.

Furthermore, Tang et al. [90] developed an electrochemical immunosensor for the detection of EGFR. This sensor was fabricated on a gold electrode surface that was first modified with SiO_2_ nanospheres through a self-assembly process. The antibody was then immobilized onto the SiO_2_ nanosphere-modified electrode, where it formed a sandwich-type immunocomplex with the EGFR antigen and a biotin-tagged nucleic acid aptamer. Subsequently, an affinity-conjugated alkaline phosphatase was attached to the electrode, and an electroactive silver layer was generated by enzymatic deposition, providing signal amplification. Experimental results showed good linearity for EGFR concentrations ranging from 1 to 1000 ng mL^−1^ and demonstrated a very low LOD of 0.06 ng mL^−1^. In conclusion, the proposed electrochemical immunosensor offers a highly sensitive and stable method for detecting EGFR as an interesting tool for clinical diagnostics.

HER2 is a protooncogene that belongs to the cell surface receptor tyrosine kinase family, which is an important cancer biomarker in the biological behavior and pathogenesis of breast cancer. Liu et al. [91] presented a novel nanosensor for the detection of breast cancer biomarkers, specifically HER2, by combining a copper-based metal–organic framework (Cu-MOF) with magnetic beads. The sensor’s construction was based on a magnetic enrichment and separation strategy. The porous structure of the Cu-MOF was used to adsorb a large number of aptamers that are designed to capture the target antigens specifically. Subsequently, magnetic beads (MB), which are modified with the corresponding antibodies, are used to enrich and separate the Cu-MOF@antigen complexes. The detection range for the HER2 biomarker was found to be 4.5 fg mL^−1^–20 ng mL^−1^ with a LOD of 1.51 fg mL^−1^.

Furthermore, the sensor showed good performance in detecting biomarkers in real human serum samples, with high recovery rates ranging from 89.00% to 107.57%. These results underscore the sensor’s high sensitivity and practical applicability. In conclusion, the developed nanosensor provides a promising solution for the detection of breast cancer biomarkers.

Also, Ai et al. [8] developed a novel photoelectrochemical (PEC) aptasensor for the ultrasensitive detection of the biomarker HER-2. The sensor’s construction involved several key components. The photoactive Z-scheme UiO-66/CdIn2S4 heterojunction was synthesized using a hydrothermal method. This material serves as the photoanode and is designed to improve photoelectric conversion efficiency by preventing the recombination of photo-generated electron-hole pairs. The second component is a flower-like PtPdCu nanozyme, fabricated via a wet-chemical method, which acts as a peroxidase-mimicking catalyst. The aptasensor is completed by immobilizing capture DNA and the PtPdCu nanozyme-modified aptamer onto the heterojunction-coated electrode. This leads to a broad linear detection range from 0.1 pg mL^−1^ to 0.1 μg mL^−1^. The sensor also achieved an impressively low LOD of 0.07 pg mL^−1^, confirming its high sensitivity. This research successfully developed a new and highly sensitive PEC aptasensor for the quantitative determination of HER-2 in human serum samples.

Moreover, Zhang et al. [92] developed an innovative electrochemical biosensor for the sensitive detection of HER2. This sensor was constructed using a combination of an aptamer (Apt), peptide nucleic acid (PNA), and magnetic Fe_3_O_4_/α-Fe_2_O_3_ heterogeneous nanorods. The mechanism involves Apt capturing the large HER2 protein, which simultaneously releases single-stranded DNA (ssDNA) chains from a double-stranded DNA (dsDNA) complex. The PNA then captures these released ssDNA chains, which converts the change in the electrochemical signal from a steric hindrance effect to one caused by the number of ssDNA strands, effectively extending the detection range. The use of magnetic Fe_3_O_4_/α-Fe_2_O_3_ nanorods combined with Apt and PNA resulted in an ultra-low LOD of 4.1 fg mL^−1^. The sensor also showed a wide detection range, from 10 fg mL^−1^ to 5 × 10^6^ fg mL^−1^. The experimental results confirmed that the biosensor has excellent selectivity, reproducibility, and storage stability. Furthermore, the analysis of spiked serum samples demonstrated a high recovery rate of 95.9–115.7%, indicating its great promise for use in real serum samples. In conclusion, this research successfully developed a highly sensitive and reliable electrochemical biosensor for HER2 detection.

CLDN18.2 belongs to the claudin family of tight junction proteins and has emerged as an attractive biomarker for gastric cancer (GC) immunotherapy. Although it is normally confined to gastric mucosal epithelial cells, its aberrant overexpression is closely associated with tumor development and metastatic progression. A study presented an electrochemical immunosensor applied to GC diagnosis by developing an electrochemical immunosensor for detecting the CLDN18.2 biomarker [93]. The study conducted a comparative analysis to determine the best material for the biosensor’s construction, exploring the deposition of polymelamine (PM) on a variety of carbon nanomaterial-based SPEs, including carbon (C), graphene oxide (GO), graphene (Gr), and carbon nanotubes (CNT). The results demonstrated that graphene and carbon nanotubes are superior substrates for the polymerization of melamine, showing more significant and stable redox behavior compared to C and GO. The immunosensors were fabricated on the PM-modified Gr and CNT platforms, which showed outstanding analytical performance. For the PM-Gr/SPE immunosensor, a linear response was obtained over the concentration range of 0.1–100 ng mL^−1^, whereas for the PM-CNT/SPE immunosensor the linear range extended from 0.01 to 100 ng mL^−1^. LODs were achieved at 7.9 pg mL^−1^ and 0.104 ng mL^−1^ for the CNT and Gr-based sensors, respectively, highlighting the platform’s high sensitivity. This superior performance is attributed to the enhanced and stable redox activity of PM on the Gr and CNT electrodes, which acts as an in situ signal transducer. This study not only introduced the electrochemical immunosensor for the CLDN18.2 protein but also established a robust methodology for the selection and modification of carbon nanomaterials.

Pepsinogen (PG) is an important biomarker of gastric digestive function and has become a useful tool in the biochemical diagnosis of GC. Two immunogenic isoforms are recognized: PG I, produced by mucous cells in the gastric fundus, and PG II, synthesized predominantly by mucous cells in the cardiac region and pyloric glands of the antrum. Clinical studies indicate that patients in early stages of GC frequently exhibit increased serum levels of these biomarkers, particularly PG I, with reported concentrations close to 70.95 μg L^−1^ and a PG I/PG II ratio of approximately 2.99 μg L^−1^. In this sense, Kanagavalli and Eissa [94] reported a redox probe-free electrochemical immunosensor for the detection of the PG I biomarker. The use of a polymelamine (PM) electrodeposited on a reduced graphene oxide (rGO) as an in situ active substrate distinguishes this study from other methods that rely on external redox probes, which simplifies the detection process and makes it more suitable for point-of-care (POC) diagnostics. The electrochemical reduction of graphene oxide (GO) on the screen-printed electrode (SPE) generated a defect-rich surface favorable for the deposition of polymelamine (PM). PG I detection was based on monitoring changes in the PM current signal. The formation of the immune complex between the immobilized anti-PG I antibody and the PG I antigen on the electrode surface caused a significant decrease in the DPV peak current. The biosensor demonstrated a clinically relevant linear concentration range of 0.01 to 200 ng mL^−1^, with a low LOD of 9.1 pg mL^−1^. This work not only presented the development of a label-free, redox probe-free electrochemical immunosensor for the GC biomarker PG I but also established a robust and promising platform for future clinical applications.

Other work presented an integrated, portable, dual-mode electrochemical immunosensing platform capable of simultaneously detecting two GC biomarkers: PG I and PG II [95]. This platform utilizes SPEs with dual working electrodes, each tailored for the detection of a specific biomarker. The electrode surface is coated with nanocomposites of reduced graphene oxide (rGO), tetraethylene pentaamine (TEPA), iron oxide (Fe_3_O_4_) nanoparticles, and bimetallic AuPt nanoparticles (AuPt NPs). The system provides linear detection ranges from 5 pg mL^−1^ to 100 ng mL^−1^ for PG I and from 50 pg mL^−1^ to 200 ng mL^−1^ for PG II. The LOD was established at 1.67 pg mL^−1^ for PG I and 16.67 pg mL^−1^ for PG II. This interesting immunosensor showed minimal cross-interference between the two working electrodes, confirming its ability to perform accurate and simultaneous quantitative measurements for GC diagnosis.

NSE is a multifunctional protein that mainly exists in nerve tissue and neuroendocrine tissue, and it is one of the important biomarkers for the diagnosis of small-cell lung cancer (SCLC). Chen et al. [96] developed an ultrasensitive photoelectrochemical (PEC) immunosensor for the NSE determination. The core element of this biosensor is a novel heterojunction of cadmium indium sulfide and magnesium indium sulfide (CdIn_2_S_4_/MgIn_2_S_4_, CMIS). The mechanism of the immunosensor is a “signal-off” system. A capture antibody (Ab1) was immobilized on the surface of the electrode modified with CMIS and platinum nanoparticles (Pt NPs). The presence of the target NSE led to the formation of a sandwich complex with a detection antibody (Ab2) attached to iron oxide nanoparticles (Fe_3_O_4_). The Fe_3_O_4_-Ab2 acts as a signal quencher, competitively consuming electron donors and absorbing light, which results in a decrease in the photocurrent. This strategy enabled the ultrasensitive detection of NSE over a wide range, from 1.0 fg mL^−1^ to 10 ng mL^−1^, with an exceptionally low LOD of 0.34 fg mL^−1^. This work provides a new perspective for the design of highly sensitive PEC immunosensors for SCLC diagnosis.

The field is rapidly advancing with new materials and designs. For liver cancer, two different approaches for detecting alpha-fetoprotein (AFP) illustrate how material choice directly impacts performance. A nanocomposite of few-layer graphene (FLG) and 3-polythiophene acetic acid (3-PTAA) provided a cost-effective and stable platform with a low LOD of 0.047 pg mL^−1^ [12]. In contrast, hollow PtCoNi bunched nanochains significantly boosted electrocatalytic activity, achieving an even lower detection limit of 0.017 pg mL^−1^ and improved long-term stability [97]. This contrast underscores the trade-off between affordability and maximum sensitivity. This comparison illustrates how different nanomaterials can be tailored for enhanced performance. Since the clinical reference cut-off for AFP is ~20 ng mL^−1^, these detection limits in the pg mL^−1^ range are several orders of magnitude more sensitive than required, ensuring applicability for early diagnosis.

**Figure 10:**
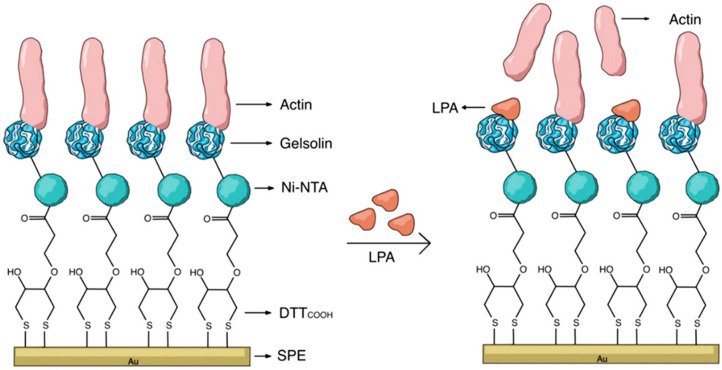
Schematic representation of the label-free electrochemical biosensor for LPA detection. Reproduce from reference Ivanova et al. [98] under the terms and conditions of the Creative Commons Attribution (CC BY) license.

For ovarian cancer, two distinct biomarkers, HE4 and LPA, have been targeted using different strategies. The disposable HE4 biosensor on a flexible ITO-PET sheet emphasizes affordability and stability for early diagnosis [16]. In contrast, the label-free LPA biosensor, based on a gelsolin–actin complex, simplifies fabrication and aligns better with point-of-care testing (Figure 10) [98]. Together, these examples highlight the dual focus of current research: ensuring both cost-effectiveness and clinical practicality. Importantly, the clinical threshold for HE4 is approximately 70 pmol L^−1^ (~140 pM), and reported biosensor sensitivities fall well below this cut-off, supporting their clinical applicability.

For colorectal cancer, a dual immunoplatform on magnetic microsupports was developed to simultaneously detect TIMP-1 and GDF-15, providing a fast and reliable method for analyzing complex clinical samples [11]. Finally, for non-small cell lung cancer, an immunosensor for the CYFRA21-1 antigen utilizes a three-dimensional ordered macroporous carbon framework (3D MCF) with gold-cobalt nanoparticles. This architecture maximizes surface area and electron transport, resulting in a highly sensitive and stable platform suitable for human serum analysis [99]. Considering that the clinical cut-off value for CYFRA21-1 is around 3–3.5 ng mL^−1^, the reported biosensor performance, with detection limits below this threshold, confirms its clinical relevance. Table 7 summarizes electrochemical biosensors for the determination of other epithelial biomarkers.

## 4. Conclusions

Epithelial cancer remains a significant global health concern, with early diagnosis being crucial for improving treatment outcomes and survival rates. Traditional methods like ELISA have notable limitations, but innovative electrochemical biosensors are emerging as a promising solution. These devices offer high sensitivity, specificity, and rapid analysis, making them ideal for cancer applications. Biosensing platforms have already been successfully established for a variety of clinically relevant biomarkers, such as CA125, CEA, and PSA, reaching low detection limits compatible with diagnostic applications. In parallel, electrochemical nucleic acid sensors, aptamer-based devices, and other advanced architectures are being engineered for the highly sensitive detection of cells and extracellular vesicles (EVs).

In this review, we have showcased the successful development of advanced electrochemical biosensors based on nanomaterials for determining epithelial cancer biomarkers in patient samples. These sensors are excellent candidates for clinical use, particularly for point-of-care testing. Their capacity to support the early detection of primary tumors, metastatic lesions, and disease recurrence significantly enhances the effectiveness of cancer management. In addition, the integration of biosensing technologies with microfluidic systems has yielded low-cost, versatile, and high-performance diagnostic platforms. These systems are attracting considerable interest owing to their high selectivity, economic feasibility, and straightforward data readout. We believe that the biggest challenge lies in developing biosensors capable of simultaneously detecting multiple biomarkers in a single measurement. This goal can be achieved through the integration of analytical chemistry and nanotechnology.

## 5. Key Challenges and Prospects

The development of electrochemical biosensors for oncology is a key technological frontier in precision medicine, promising early detection and personalized treatment monitoring. These miniaturized devices, which offer high sensitivity and specificity, must overcome substantial barriers for widespread Point-of-Care Testing (POCT) adoption, ranging from materials engineering and complex sample management to rigorous clinical validation.

One of the primary impediments to commercialization is the long-term stability of the bio-recognition elements (such as antibodies and aptamers), which are inherently fragile and susceptible to degradation. This limited functional lifespan imposes severe restrictions on storage and usability. Addressing this requires significant investment in optimizing and characterizing modified electrodes to ensure their robustness under diverse environmental conditions.

Closely related to stability is the challenge of batch manufacturing and batch-to-batch reproducibility. Although techniques like screen-printing (SPEs) enable automated, low-cost mass production, variability in the biofunctionalization of electrodes with nanomaterials affects sensitivity uniformity. Overcoming this quality control barrier is essential for reducing production costs and justifying automation investment, which is a fundamental requirement for regulatory certification.

At the analytical level, biosensors must struggle with the matrix effect of clinical samples. Body fluids like serum are complex matrices with high concentrations of interfering species. These interferents generate a background signal that compromises the device’s selectivity and precision. A key strategy to mitigate this is the development of multisensor systems or “electronic tongues” that, when combined with pattern recognition software, can effectively analyze such complex samples.

A further analytical obstacle is the detection of low-concentration cancer biomarkers in early stages, an essential requirement for liquid biopsy. For example, the trace concentration of methylated DNA (as low as 25 fg mL^−1^) demands extreme analytical sensitivity, surpassing conventional methods by up to a thousand times. This underscores the critical need for active analytical platforms that allow for the cancer biomarker sample pre-concentration before detection.

In the clinical context, validation requires rigorous testing in representative cohorts that include healthy subjects, patients with premalignant lesions, and cancer at various stages. The need for standardization is imperative, as multiplexed molecular biosensors must achieve a level of reliability comparable to histopathological gold standards and comply with regulatory recommendations, such as those from the FDA, particularly for liquid biopsy applications.

Microfluidics and Lab-on-a-Chip (LoC) systems are a key technological perspective, as they automate the management of small sample volumes and significantly reduce analysis time. Their most significant impact lies in their ability to isolate and enrich trace biomarkers from liquid biopsy (e.g., circulating tumor cells, exosomes, circulating tumor DNA). LoC systems fundamentally transform the biosensor from a simple detector into a complete pre-treatment and analysis system.

From a hardware perspective, flexible electrodes and paper-based devices (for wearables and POCT) represent the most promising direction. Screen-printed electrodes enable low-cost production and design flexibility, facilitating multiplexed detection. This technology is fundamental for the continuous monitoring of physiological and chemical signals in the comprehensive management of the oncological patient.

Artificial Intelligence and Machine Learning are essential for processing the complex data generated by multiplexed biosensors. AI not only processes large amounts of medical data but also optimizes the signal by eliminating noise and interference generated by movement or the environment in real time, a capability critical for flexible hardware and wearable devices.

Finally, AI is transforming clinical decision support, offering early diagnosis with high accuracy and the ability to predict treatment response. The ultimate future perspective, however, is the evolution toward prognostic prediction, the ability to differentiate between non-cancerous changes that resemble cancer from truly aggressive pathologies, thus mitigating the risk of over-diagnosis and unnecessary treatments.

## Figures and Tables

**Table 2:** Electrochemical biosensors for CEA determination.

Nanomaterial	Biosensor Type	Linear Range	Detection Limit	Sample	Ref.
4-mercaptophenyl /AuNPs	Aptasensor	0.5–10 ng mL^−1^	0.102 ng mL^−1^	Serum	[7]
ZrO_2_-AuNPs	Aptasensor	0.01–104 pg mL^−1^	42.504 fg mL^−1^	Serum	[42]
Fc/PdPt@PCN-224	Aptasensor	1 pg mL^−1^–100 ng mL^−1^	0.27 pg mL^−1^	Serum	[19]
Au@MoS_2_@rGO	Aptasensor	0.1 pg mL^−1^–100 ng mL^−1^	0.019 pg mL^−1^	Serum	[44]
IDE	Aptasensor	0.002 ng mL^−1^– 2 ng mL^−1^	3.8 pg mL^−1^	Serum	[43]
MNC	Immunosensor	500 fM–50 nM	500 fM	Serum	[38]
Au-MoO_3_-Chi	Immunosensor	0.001–0.01 ng mL^−1^	0.5 pg mL^−1^	Serum	[35]
CDs	Immunosensor	0.5–50 ng mL^−1^	10 pg mL^−1^	Serum	[39]
Fe_3_O_4_/MWCNTs	Immunosensor	0.005–2.5 ng mL^−1^	0.3 pg mL^−1^	Serum	[37]
MoO_3_-rGO-IL	Immunosensor	25 fg mL^−1^–100 ng mL^−1^	1.19 fg mL^−1^	Serum	[36]
AuNPs	Immunosensor	0.1–40 ng mL^−1^	0.03 ng mL^−1^	Serum	[45]
VMSF	Immunosensor	0.01 ng mL^−1^–100 ng mL^−1^	6.3 pg mL^−1^	Serum	[46]
ZnONPs/GNPs	Immunosensor	0.5–10.0 ng mL^−1^	0.44 ng mL^−1^	Serum	[40]
MX@CNT	Immunosensor	0.005–1.0 ng mL^−1^	1.6 pg mL^−1^	Serum	[41]

Note: Au-MoO_3_-Chi: gold nanoparticles-molybdenum trioxide-chitosan; AuNPs: gold nanoparticles; FcPdPt@PCN-224: ferrocene PdPt nanoparticles@Zr-based porphyrinic metal–organic framework; Au@MoS_2_@rGO: gold@disulfide@graphene oxide; MNC: nitrogen-rich mesoporous carbon; CDs: carbon dots; Fe_3_O_4_: iron oxide nanoparticles; MWCNTs: multi-walled carbon nanotubes; MoO_3_-rGO-IL: ionic liquid-functionalized molybdenum trioxide-reduced graphene oxide; ZrO_2_-AuNPs: zirconia-gold nanoparticles; IDE: interdigitated gold electrode; VMSF: ordered mesoporous silica film; ZnONPs: flower-like zinc oxide nanoparticles; GNPs: graphene nanoplatelets; MX@CNT: carbon nanotube-bridged Ti_3_C_2_Tx MXene.

**Table 3:** Electrochemical biosensors for PSA determination.

Nanomaterial	Biosensor Type	Linear Range	Detection Limit	Sample	Ref.
AuNPs/2D-MoS_2_	Immunosensor	0–10 ng mL^−1^	3.58 pg mL^−1^	Serum	[47]
AuNPs	Immunosensor	0.1–50,000 pg mL^−1^	0.083 pg mL^−1^	Serum	[48]
CDs@PANI	Immunosensor	0.01–60 ng mL^−1^	20 pg mL^−1^	Serum	[49]
AuNPs	Immunosensor	10–104 pg mL^−1^	102 fg mL^−1^	Serum	[51]
GQDs	Immunosensor	0.0125–1.0 ng mL^−1^	0.005 ng mL^−1^	Serum	[56]
2FcP-GA-GDY(Fe)@NMIL-B	Peptide biosensor	10 fg mL^−1–^50 ng mL^−1^	0.94 fg mL^−1^	Serum	[52]
AuNPs	Protein biosensor	0.01–100 ng mL^−1^	3.574 pg mL^−1^	Serum	[53]
Nanopore/MIPs	Aptasensor	5–100 ng mL^−1^	0.42 ng mL^−1^	Serum	[50]
rGO/g-C_3_N_4_/AuNPs	Aptasensor	2.5–12.5 pM	0.44 fM	Serum	[54]
α-Fe_2_O_3_/Fe_3_O_4_@Au	Aptasensor	100 fg mL^−1–^100 ng mL^−1^	0.78 pg mL^−1^	Serum	[55]

Note: AuNPs/2D-MoS_2_: gold nanoparticles/two-dimensional molybdenum disulfide; CDs@PANI: carbon dots functionalized polyaniline; MIPs: molecularly imprinted polymers; 2FcP-GA-GDY(Fe)@NMIL-B: Fe-Graphdiyne into a metal–organic framework material NH2-MIL88B(Fe); rGO/g-C_3_N_4_: reduced graphene oxide/graphitic carbon nitride; α-Fe_2_O_3_/Fe_3_O_4_@Au: magnetic nanoparticles/gold nanoparticles; GQDs: Graphene quantum dots.

**Table 4:** Electrochemical biosensors for miRNA determination.

Nanomaterial	Biosensor Type	Linear Range	Detection Limit	Sample	Ref.
AuNRs-AuNPs	Aptasensor	0.1 fM–100 pM	5.10 aM/9.39 aM	Serum	[57]
PNA-DNA H3WJ	PNA	0.5 fM–5 nM	0.15 fM	Serum	[58]
AZO nanostars	DNA	1 pM–10 nM	3.98 pM	Breast cells	[59]
PER-CRISPR	DNA	10−13–10−7 M	30.2 fM	Serum	[60]
GO + G	DNA	10 fM–1 nM	3.18 fM	Serum	[61]
AuNPs	DNA	0.1–1000 nM	1 nM	Serum	[62]
Au/MWCNT	DNA	0.001–10 nM	0.73 pM	Serum	[63]
AuNPs	DNA	1–100 nM	50 pM	Serum	[64]
Lentil lectin (LCA)-MB	DNA	1 fM–10 pM	26 aM	Serum	[65]
IONPs	DNA	0.1 pM–1µM	0.023 pM	Serum	[66]
AuNPs/MBs/COFs	DNA	10 fM–5 nM	1.2 fM	Serum	[67]
PNA	PNA	0.1 fM–0.1 nM	12.4 aM	Serum	[68]
AuNPs	DNA	~1–100 nM	0.4 nM	Serum	[69]
PNA-MXene (Ti_3_C_2_Tx)	PNA	100 aM–10 nM	40 aM	Serum	[70]
PER-CRISPR	DNA	—	0.43 fM/0.12 fM	Serum	[71]
AuNPs	DNA	10 aM–100 pM	5.24 aM	Plasma	[72]
GO	DNA	0.1 pM –10 nM	0.029 pM	Serum	[73]
LIG+PNA	PNA	100 aM–100 nM	0.6 aM	Serum	[74]
PB-COFs nanospheres	DNA	10 fM–100 nM	2.5 fM	Serum	[75]

Note: AuNPs: gold nanoparticles; AuNRs: gold nanorods; AZO: aluminum-doped zinc oxide; COFs: covalent organic frameworks; G: Graphene; GO: Graphene oxide; H3WJ: hetero-three-way junction; IONPs: Iron oxide nanoparticles; Au/MWCNT: gold/multiwall carbon nanotube; NAL: nucleic acid lock; PB: Prussian Blue; PER: primer exchange reaction; PNA: peptide nucleic acids.

**Table 5:** Electrochemical biosensors for Extracellular Vesicles determination.

Nanomaterial	Biosensor Type	Linear Range	Detection Limit	Sample	Ref.
GOxS1 (DNAzyme)	Aptasensor	3.63 × 10^4^–7.26 × 10^8^ particles mL^−1^	3.63 × 104 particles mL^−1^	Plasma	[76]
MCH–AuNPs	Aptasensor	88–8.8 × 10^7^ particles μL^−1^	22 particles μL^−1^	Serum	[81]
MB-DNA probes	DNA	1 × 10^2^–1 × 10^8^ aM	45 aM	Exosomal miRNA	[78]
PbS CQDs	Immunosensor	10^2^–10^8^ particles mL^−1^	19 particles mL^−1^	Serum	[77]
GO + AuNPs	Immunosensor	500–1 × 10^7^ exo μL^−1^	110 exo μL^−1^	Serum	[79]
AuNPs	Immunosensor	10^5^–10^12^ exo mL^−1^	8.7 × 10^2^ exo mL^−1^	Urine	[80]

Note: AuNPs: gold nanoparticles; CQDs: colloidal quantum dots; GO: Graphene oxide; GOx-S1: glucose oxidase-substrate strand S1; MB-DNA: methylene blue—DNA; MCH: 6-mercapto-1-hexanol.

**Table 6:** Electrochemical biosensors for DNA/Tumor-Cell/ctDNA determination.

Nanomaterial	Biosensor Type	Linear Range	Detection Limit	Sample	Ref.
Fe-MOF	Cytosensor	10–10^5^ (EC)/150–10^5^ (CL)	3 cells mL^−1^ (EC)/10 cells mL^−1^ (CL)	HepG2 cells	[17]
AuNPs	Aptasensor	100–10^6^ cells/mL	21 cells mL^−1^	MUC1^+^ cells	[82]
Ni@MWNT	Immunosensor	10^2^–10^7^ cells/mL	2 cells mL^−1^	Mouse liver tissue	[83]
rGO + IONPs	Immunosensor	10^2^–10^6^ cells/mL	5 cells mL^−1^	SKBR3 cells	[84]
AuNPs	DNA	1 pM–50 pM	178 fM/216 fM	Mouse serum	[85]
(Au-Pt–Ag)-PDMS	DNA	2–200 fM	24.1 fM	Carcinoma cells	[86]
Au@Fe_3_O_4_NPs	DNA	—	~3 aM	Plasma	[87]

Note: AuNPs: gold nanoparticles; Fe-MOF: Metal–Organic Frameworks; IONPs: Iron oxide nanoparticles; Ni@MWNT: Ni-coated multi-walled carbon nanotube; PDMS: polydimethylsiloxane; rGO: reduced Graphene oxide.

**Table 7:** Electrochemical biosensors for the determination of other epithelial biomarkers.

Nanomaterial	Biomarker Type	Biosensor Type	Linear Range	Detection Limit	Sample	Ref.
Fe_3_O_4_/α-Fe_2_O_3_@Au	EGFR	Aptasensor	0.1–1000 ng mL^−1^	0.18 ng mL^−1^	Serum	[88]
Magnetic NPs	EGFR	Aptasensor	10 fM–1 µM	372 aM	Serum	[89]
Fe_3_O_4_/α-Fe_2_O_3_	HER 2	Aptasensor	10 fg mL^−1^–5 × 10^6^ fg mL^−1^	4.1 fg mL^−1^	Serum	[92]
DTTCOOH	LPA	Biosensor	0.01–10 μM	0.9 µM	Serum	[98]
SiO_2_NPs	EGFR	Immunosensor	1–1000 ng mL^−1^	0.06 ng mL^−1^	Serum	[90]
Gr/CNT	CLDN18.2	Immunosensor	0.1–100 ng mL^−1^ and 0.01 ng mL^−1^–100 ng mL^−1^	7.9 pg mL^−1^ for CNT and 0.104 ng mL^−1^ for Gr	Serum	[93]
GO	PG I	Immunosensor	0.01–200 ng mL^−1^	9.1 pg mL^−1^	Serum	[94]
rGO/Fe_3_O_4_NPs/AuPtNPs	PGI/PGII	Immunosensor	5 pg mL^−1^–100 ng mL^−1^ for PG I and 50 pg mL^−1^–200 ng mL^−1^ for PG II	1.67 pg mL^−1^ for PG I and 16.67 pg mL^−1^ for PG II	Serum	[95]
PtNPs/Fe_3_O_4_NPs	NSE	Immunosensor	1.0 fg mL^−1^–10 ng mL^−1^	0.34 fg mL^−1^	Serum	[96]
3-PTAA/FLG	AFP	Immunosensor	0.0001–250 ng mL^−1^	0.047 pg mL^−1^	Serum	[12]
GH-PtCoNi BNCs	AFP	Immunosensor	0.1 to 10^5^ pg mL^−1^	0.017 pg mL^−1^	Serum	[97]
ITO-PET/3-APTES	HE4	Immunosensor	1 pg/mL to 3000 pg mL^−1^	0.094 pg mL^−1^	Serum	[16]
HOOC-MBs	TIMP-1/GDF-15	Immunosensor	43.4–2500 pg mL^−1^	13 pg mL^−1^	Plasma	[11]
Au/CoNPs-3D MCF	CYFRA21-1	Immunosensor	0.0001–100 ng mL^−1^	0.0224 pg mL^−1^	Serum	[99]

Note: Fe_3_O_4_/α-Fe_2_O_3_: Magnetic nanoparticles; Au: Gold nanoparticles; NPs: nanoparticles. MOF: metal–organic framework; Gr: graphene; CNT: carbon nanotubes; GO: graphene oxide; rGO: reduced graphene oxide; 3-PTAA: 3-polythiophene acetic acid; FLG: few-layer graphene; GH-PtCoNi BNCs: gourd-shaped hollow PtCoNi bunched nanochains; ITO-PET: indium tin oxide-polyethylene terephthalate sheet; 3-APTES: 3-Aminopropyl trimethoxy silane; DTTCOOH: 3-dithiothreitol propanoic acid; HOOC-MBs: magnetic beads; Au/CoNPs-3D MCF: macroporous carbon skeleton material modified with gold-cobalt nanoparticles.
